# Extinction risk to lake minnow (*Eupallasella percnurus*) due to habitat loss: Eastern Poland case study

**DOI:** 10.1007/s10661-019-7731-6

**Published:** 2019-08-16

**Authors:** Barbara Sowińska-Świerkosz, Marcin Kolejko

**Affiliations:** 1 Department of Hydrobiology and Protection of Ecosystems, University of Life Sciences in Lublin, Dobrzańskiego 37, 20-262 Lublin, Poland; 2 PGW WP - State Water Holding Polish Waters, The Regional Water Management Authority in Lublin, Leszka Czarnego 3, 20-610 Lublin, Poland

**Keywords:** Eastern Poland, Extension risk, Habitat loss, Lake minnow, Peat holes

## Abstract

In Poland, lake minnow (*Eupallasella percnurus* Pall.) inhabit 160 sites, including 44 in the eastern part of the country. Their habitats are mainly small and shallow peat holes vulnerable to complete destruction due to being dried or overgrown. Such processes are regularly observed, with ~ 60% of sites having vanished since the 1950s. Therefore, this species is one of the most endangered fish species from the *Cyprinidae* family in Polish inland waters. A GIS approach was adopted to fully analyze their habitat loss. The habitat area was marked in four time periods (from the 1960s to 2018) in reference to 26 sites composed of 111 reservoirs and representing almost 60% of all those documented in Eastern Poland. On this basis, the rate at which the habitat will vanish and the predicted time when this will happen were calculated. The results showed that the mean vanishing rate oscillates between 1 and 2% of the area per year for 50% of the analyzed sites (*N* = 13). For three sites (11%), this value does not exceed 1% of each area per year and is higher than 2% for the rest of the sites (39%). The results indicate that if the process of overgrowing and shallowing is not stopped, 58% of the analyzed sites will disappear in the next 50 years (including 8 (31%) in the next 20 years). This trend may lead to a serious decline in the species population or even its extinction in the next decades. Passive protection has proven to be insufficient in preserving lake minnow habitats. Therefore, there is an urgent need to undertake decisive protection action, as proposed in this manuscript.

**Electronic supplementary material:**

The online version of this article (10.1007/s10661-019-7731-6) contains supplementary material, which is available to authorized users.

## Introduction

Both natural processes and human land-use activities may have contributed to the native habitat loss and thus to the local and global extinction of the fish species (Wilcove et al. 1998). Guidelines for assessing extinction risk have been proposed by the International Union for Conservation of Nature (IUCN). However, the assessment criteria used by the system (IUCN 2001) are difficult to apply to fishes (Zhang et al. 2015). The scientists have started to establish alternative criteria that can be divided into two groups. The first group is related to population reduction (e.g., reproductive potential, the actual or potential levels of exploitation, the effects of taxa introduced, hybridization, pathogens, pollutants, competitors, or parasites). The other one is associated with the geographic range (size/fluctuation of the area/extent of occupancy) (Baigún et al. 2012; Keith and Marion 2002). There is still no comprehensive and unifying set of criteria for estimating extinction of fishes on a global scale (Duncan and Lockwood 2011). The main factors affecting the extinction risk of fishes are climate change (Cheung et al. 2018), alien species (Costantini et al. 2018), and land-use changes leading to habitat loss (Fu et al. 2018; Glamuzina et al. 2018; Spens et al. 2007). In relation to the latter factor, the commonly used ecological model concerning this issue is the species–area relationship (SAR) derived from MacArthur and Wilson’s (1963, 1967) theory of island biogeography. Many other models have followed (Kadmon and Allouche 2007, Rosindell and Cornell 2007; Whittaker et al. 2008). Despite the differences in the proposed SAR models, they are commonly based on the mutual relationship between the number of species and the area of habitat. Additional parameters are used habitat heterogeneity, probability of population survival, population size, the spatial configuration of the habitat, and the way species are sampled (Rybick and Hanski 2013). Generally, the SAR model shows three phases depending on the research scale. A relatively steep slope is typical of very small areas with low numbers of individuals per species. As for regional scales, a shallower slope can be identified, and as far as very large scales with different sets of species are concerned, a steep slope can be noticed (Hubbell 2001). Many researchers state that this model overestimates extinction as environmental and ecological variables are the same before and after habitat loss. A more elaborated model is called the endemics–area relationship (EAR). It predicts the number of species becoming extinct instantly after their habitat has vanished, but does not take into account that usually many other species become extinct soon afterwards because habitat conditions in the remaining habitat have changed (Kinzig and Harte 2000).

Those and many other models focus on landscape fragmentation as a major factor influence the population size and state. However, similar land-use patterns may influence the status and long-term viability of populations within these landscapes differently. For example, if the landscape transformations are faster than the demographic response time of the species, populations may respond to habitat loss with a delay (i.e., extinction debt) (Hanski and Ovaskainen 2002). A population which seems to be initially unaffected by landscape disturbance may show a decline after years or decades later even after the changes have stopped. Assessment of extinction risk depends not only upon the current state of the landscape and its projected transformations but also on its past disturbance history (Schrott et al. 2005). Consequently, conservation of endangered species, including fish species, must be done within a dynamic landscape context, taking into account both natural processes and human land-use activities. Projections based on the current state of landscape will only probably have limited applicability when habitat loss is an ongoing process or if it has occurred recently (Schrott et al. 2005).

GIS techniques are widely used to assess the extension risk of fish species. These techniques are used rather as additional tools of analysis than major ones. They allow for the identification of the most important spatial parameters that influence extinction. For example, in population viability analysis (PVA), remote sensing can be used to assess different environmental indicators that affect the existence of a given population in a certain area (Leasure et al. 2019; Loannidou and O’Hanley 2019; Roberts et al. 2016). GIS techniques also allow for the identification of which habitat patches have the greatest effect on metapopulation viability and to run extinction simulations under various model conditions (McCusker et al. 2017) of executed analysis based on historical data to identified parameters that may affect fish extinction in the present (Klippel et al. 2016; Tedesco et al. 2005). They are also used in species distribution models (SDMs) to obtain habitat suitability maps based on historical species occurrences and environmental variables (Klippel at el. 2016). Remote sensing proved its usefulness in waterbody connectivity analysis to conclude on the presence–absence of a certain species based on the existence of physical barriers (Diebel et al. 2015; Muhlfeld et al. 2015; Spens et al. 2007). Such models exhibit high predictive power and have the potential to be of vital use in predicting the distribution of freshwater fish (Spens et al. 2007). Remote sensing may also be used to model causes of fish extinction, other than those derived from land-use and water fluctuation. Satellite data were applied to create maps to assess which fish species were potentially in the region of the 2010 Gulf of Mexico oil spill and to what degree their range was exposed to pollution (Chakrabarty et al. 2012).

Therefore, the manuscript aims at assessing the extinction risk of lake minnow as a result of habitat vanishing based on GIS. We applied the dynamic model, i.e., a model in which past landscape transformations are analyzed together with the current state of the habitat. The prediction is based on analysis of habitat loss processes observed in the last decades and presented as a mathematical model. This approach makes it possible to evaluate the mean time period for each population’s extinction understood as the mean time period during which a reservoir will vanish due to overgrowing and swallowing processes treated as the most probable pathway of the population on the way to extinction.

## Study site

The lake minnow (*Eupallasella percnurus* Pall.) is one of the rarest and the most endangered fish species of the *Cyprinidae* family in Polish inland waters. The species comprises small phytophilic fish, which belong to the fish family of cyprinids (*Cyprinidae*). The occurrence of this fish species is highly dispersed spatially (Kottelat and Freyhof 2007). Its global coverage stretches from the Polish part of the Odra River basin to the west of the Pacific coast of Siberia, the Kamchatka Peninsula, the island of Sakhalin (Kluchareva 1964) and Hokkaido (Shimazu 1992), China, and the Korean Peninsula to the east (Mitrofanov et al. 1987). However, the species mainly inhabit large areas in the north of Europe and Asia. The global population of lake minnow displays a wide range of morphological variation, which resulted in five distinguished subspecies (Kusznierz et al. 2017). Across Poland, however, genetic variation in this species is quite low (Kaczmarczyk and Wolnicki 2016).

**Fig. 1:**
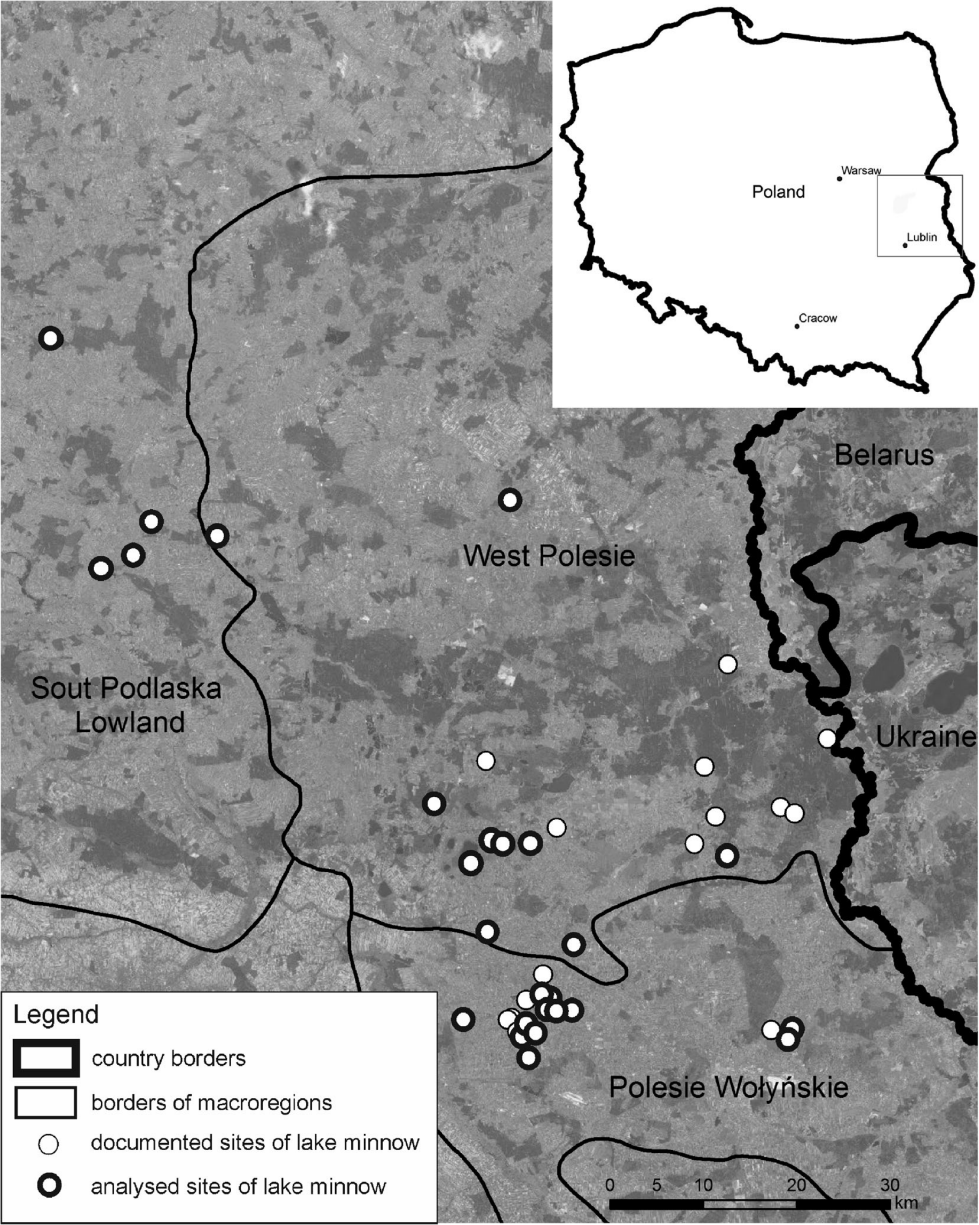
Localization of the study area and documented sites of lake minnow in Eastern Poland

In Poland, there are about 160 sites distributed non-uniformly in five voivodeships: Pomorskie, Wielkopolskie, Kujawsko-Pomorskie, Mazowieckie, and Lubelskie (Radke et al. 2011, Sikorska et al. 2011, Wolnicki et al. 2011a, b, c). The vast majority of the sites are found in the Pomorskie Voivodeship and in the Lubelskie Voivodeship (Wolnicki et al. 2011a). In the latter region situated in the east of Poland, the first comprehensive study aimed at finding lake minnow habitats was carried out between 2005 and 2008 (Wolnicki and Kolejko 2008,Wolnicki et al. 2011a). In the years 2014–2015, the inventory was repeated that confirmed the presence of lake minnow in most of them. To avoid excessively invasive methods which would increase the mortality of fish and affect their environment, particularly aquatic vegetation during the spawning season, the species population was estimated based on the number of fish caught in the trap net per unit of time (Kusznierz 2011). As a result, lake minnow occurrence was assessed in 44 sites (Wolnicki et al. 2011a). The Polesie Wołyńskie macroregion and the West Polesie macroregion have 20 sites each and the other 4 sites can be found in the South Podlaska lowland (Fig. 1).

Lake minnow in Eastern Poland inhabit peat holes—sites where peat was excavated in the late nineteenth and the first half of the twentieth century (Hliwa et al. 2017, Wolnicki and Kolejko 2008, Wolnicki and Radke 2009). They are mostly small, closed water bodies with surface areas not exceeding 0.5 ha. These water bodies are small and extremely shallow (between 0.5 and 1.5 m) making them vulnerable to complete destruction due to drying or overgrowing with plants. Repopulation of new reservoirs took place during the spring thaw when a species was moving at the small distances. The research conducted by Wolnicki and Kolejko (2008) on the Polesie Lubelskie region showed that over two dozens (30) of documented habitats have vanished in the last 60 years. This corresponds to about 60% of the total number of habitats documented in the 1960s and 1970s by Danilkiewicz (1965, 1968, 1973). This trend may lead to a serious decline in lake minnow population in decades to come or even its extinction in Eastern Poland. That is why it is vital to analyze habitats of lake minnow population fully in this region.

## Materials and methods

The method of analyzed extinction risk to lake minnow (*Eupallasella percnurus*) due to habitat loss is composed of five stages.

### Pre-analysis

Firstly, all 44 habitats (sites) of lake minnow documented in Eastern Poland were pre-analyzed (Wolnicki et al. 2011a) based on the topographic maps and orthophotomaps of the analyzed areas. Then, sites with the following characteristics were rejected: (1) those that did not exist in the 1960s and 1970s or 1980s, a period that serves as reference for analysis, and (2) those very small and heavily overgrown, invisible, or barely visible on the orthophotomap. As a result, 26 sites composed of 111 reservoirs and representing almost 60% of all those documented in Eastern Poland were included for further analysis. There are two types of such habitats: (1) single peat holes—9 sites, and (2) their complexes consisting of several to several dozen individual small reservoirs—17 sites.

### Assessing of the reservoirs’ area

Using the ArcGIS 10.3 software, the surface of the water visible was marked manually in four periods, depending on the availability of data, i.e.:

1. First period (P1): the year 1961, 1977, or 1985 (topographic map, 1:10 000);
2. Second period (P2): the year 2003 or 2004 (orthophotomap, pixel cell size of 0.25 m);
3. Third period (P3): the year 2009 or 2012 (orthophotomap, pixel cell size of 0.25 m);
4. Fourth period (P4): the year 2017 or 2018 (orthophotomap, pixel cell size of 0.25 m).

The smallest polygon marked had an area of 0.001 ha. During the work, it occurred that 35 of the analyzed reservoirs were not visible on orthophotomap 2017 (2018) (marked by * in the Supplementary Materials). Therefore, the last reference year of analysis conducted in reference to those reservoirs was selected for period P3 (2009 or 2012), dependently of the availability of data. As a result, the area of each reservoir in each analyzed year was defined

### Calculation of vanishing rate

Since the maps that served as data sources were created in different time periods and the analyzed reservoirs were of various sizes, the rate at which habitats vanish was expressed in percentages per year, rather than in hectares per year (which would make comparisons across the sites impossible). Two types of the mean rate were calculated: the mean rate (*R*_mean_), which showed the differences between the P1 and P4(P3), and the 10 or 15-year rate (*R*_15-years)_ to refer to the differences between the P2 and P4(P3). Such a methodology was adopted as a topographic map and features much lower accuracy in representing borders of small reservoirs than the orthophotomap. The *R*_mean_ gives insight into the direction and intensification of vanishing processes, wherein the last *R*_15-years_ allowed us to estimate the area of reservoir that disappeared in a given period of time. Analyses were performed both in relation to whole sites and to individual reservoirs with a given site. The following formula was used:

a $$ {R}_{\mathrm{mean}}=\frac{A_{P1-P4(P3)}}{T_{P1-P4(P3)}} $$

b $$ {R}_{15-\mathrm{years}}=\frac{A_{P2-P4(P3)}}{T_{P2-P4(P3)}} $$

where *R*_mean_—mean vanishing rate; *R*_15-years_—15-year rate; *A*—percentage of the water area disappearing between given periods of analysis (%); and *T*—difference in year between given periods of analysis.

### Estimation of the prognosis date of reservoirs vanishing

Then, we calculated when each site as well as each reservoir would disappear if the observed trend persisted. As a result, two dates were identified—based on the *R*_mean_ and *R*_15-years_. It allows estimating roughly how long it will take for lake minnow to become extinct in the east of Poland due to the loss of their habitat.

The estimation of the date of reservoirs vanishing was based on the following formula:

c $$ P{Y}_{\mathrm{mean}}= Yea{r}_{P4(P3)}+\frac{Are{a}_{P4(P3)}}{R_{\mathrm{mean}}} $$

d $$ P{Y}_{15-\mathrm{years}}= Yea{r}_{P4(P3)}+\frac{Are{a}_{P4(P3)}}{R_{15-\mathrm{years}}} $$

where PY_mean_—prognosis year of reservoir vanishing based on *R*_mean_; PY_15-years_—prognosis year of reservoir vanishing based on *R*_15-years_; Year—a given period of time; and Area—area of reservoir (m^2^) in a given period of analysis.

### Estimation of the correction factors

GIS analysis showed the intensification of negative trends leading to the reservoir vanishing between two last analyzed periods. In relation to most of the reservoirs, trends observed between P1 and P3 changed significantly after P3 resulted in a more rapid overgrowing and shallowing process. This is why the model had to be rebuilt to obtain more likely data of reservoir vanishing. Firstly, a sensitivity analysis was carried out to select the best correction factors to estimate the final data of reservoir vanishing. Different factors and factors values were analyzed to check the prediction power of the model. Finally, three factors were added to the model, which lower the prognosis year of reservoir vanishing (Table 1).

**Table 1:** Correction factors of estimating the prognosis date of reservoir vanishing

Symbol	Type	Factor	Description
F1	Depth and area in P4(P3)	0	1 m < depth < 2 m and area > 10 000 m^2^
		0.0005	1 m < depth < 2 m and 1000 m^2^ < area < 10 000 m^2^
		0.001	Depth < 1 m and 100 m^2^ < area < 1000 m^2^
			1 m < depth < 2 m and area < 100 m^2^
		0.0015	Depth < 1 m and Area < 100 m^2^
F2	Vanishing model between P3(P2) and P4(P3)	0	Shrinkage < 50% (area of reservoir in P4(P3) decreased less than 50% in relation to P3(P2))
		0.0005	Shrinkage > 50% (area of reservoir in P4(P3) decreased more than 50% in relation to P3(P2))
		0.001	Shrinkage < 50% and dissection
		0.0015	Shrinkage > 50% and dissection
F3	Land-use changes* between P3(P2) and P4(P3)	0	No changes
		0.0005	Changes < 33%**
		0.001	33%** < changes > 66%**
		0.0015	Changes > 66%**

*Land-use changes mean overgrowing of meadows ecosystems that surrounded reservoirs with shrubs and forests

**Means the % of length of reservoir borders adjoining the area of land-use changes

**Fig. 2:**
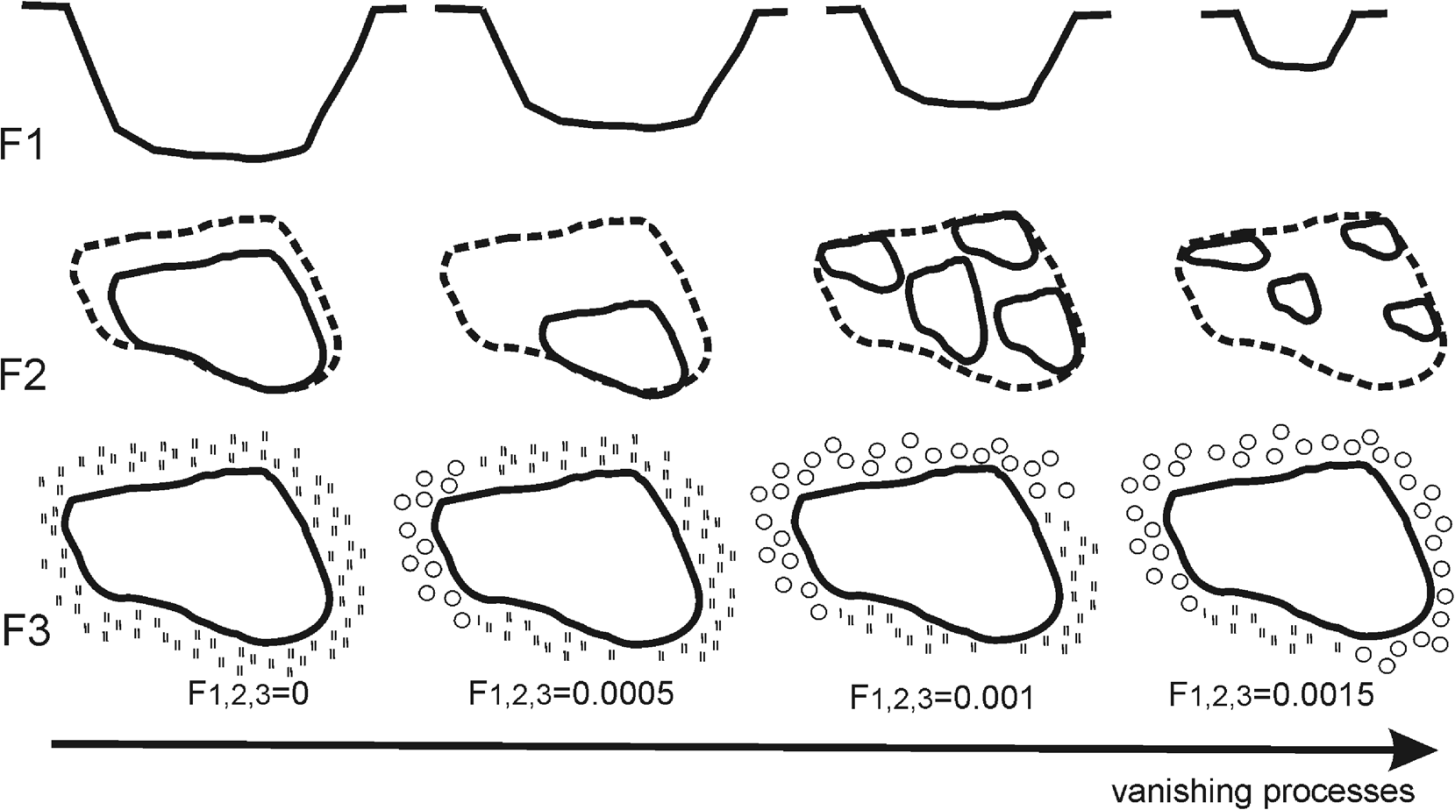
Hypothetical examples of the correction factors application

The depth and area factor was applied, since small and shallow peat bogs are supposed to vanish faster than larger and deeper ones (Fig. 2). The Vanishing Model refers to the fact that observing in the last year, dissection processes will result in more rapid overgrowing—a peat bog hole constituting one reservoir is less vulnerable to vanishing than a hole constituting many small reservoirs. Land-use changes refers to the intensification of overgrowing processes of meadows with shrubs and forests observed in the last years, which resulted in faster habitat vanishing.

As a result, the final formulas of the prognosis year of a reservoir vanishing were as follow:

e $$ \mathrm{PY}{\mathrm{Cor}}_{\mathrm{mean}}=\mathrm{P}{\mathrm{Y}}_{\mathrm{mean}}-\left(\mathrm{P}{\mathrm{Y}}_{\mathrm{mean}}\ast \mathrm{F}1+\mathrm{P}{\mathrm{Y}}_{\mathrm{mean}}\ast \mathrm{F}2+\mathrm{P}{\mathrm{Y}}_{\mathrm{mean}}\ast \mathrm{F}3\right) $$

f $$ \mathrm{PY}{\mathrm{Cor}}_{15-\mathrm{years}}=\mathrm{P}{\mathrm{Y}}_{15-\mathrm{years}}-\left(\mathrm{P}{\mathrm{Y}}_{15-\mathrm{years}}\ast \mathrm{F}1+\mathrm{P}{\mathrm{Y}}_{15-\mathrm{years}}\ast \mathrm{F}2+\mathrm{P}{\mathrm{Y}}_{15-\mathrm{years}}\ast \mathrm{F}3\right) $$

where PYCor_mean_—corrected prognosis year of reservoir vanishing based on *R*_mean_; PYCor_15-years_—corrected prognosis year of reservoir vanishing based on *R*_15-years_; *F*—correction factors according to Table 1

The *R*_mean_ and *R*_15-years_ of a site were based on the mean of rates of all individual small reservoirs constituting this site. Corrected prognosis year of a site vanishing was based on the maximum PYCor_mean_ and PYCor_15-years_ calculated for individual reservoirs consisting this site.

One-way analysis of variance (ANOVA) was executed to define the differences between *R*_mean_ and *R*_15-years_ as well as between PYCor_mean_ and PYCor_15-years_ and in relation to each group of sites determined based on their geographical location. Statistical differences between means were measured using the Tukey post hoc tests.

## Results

### Models of reservoir vanishing

**Fig. 3:**
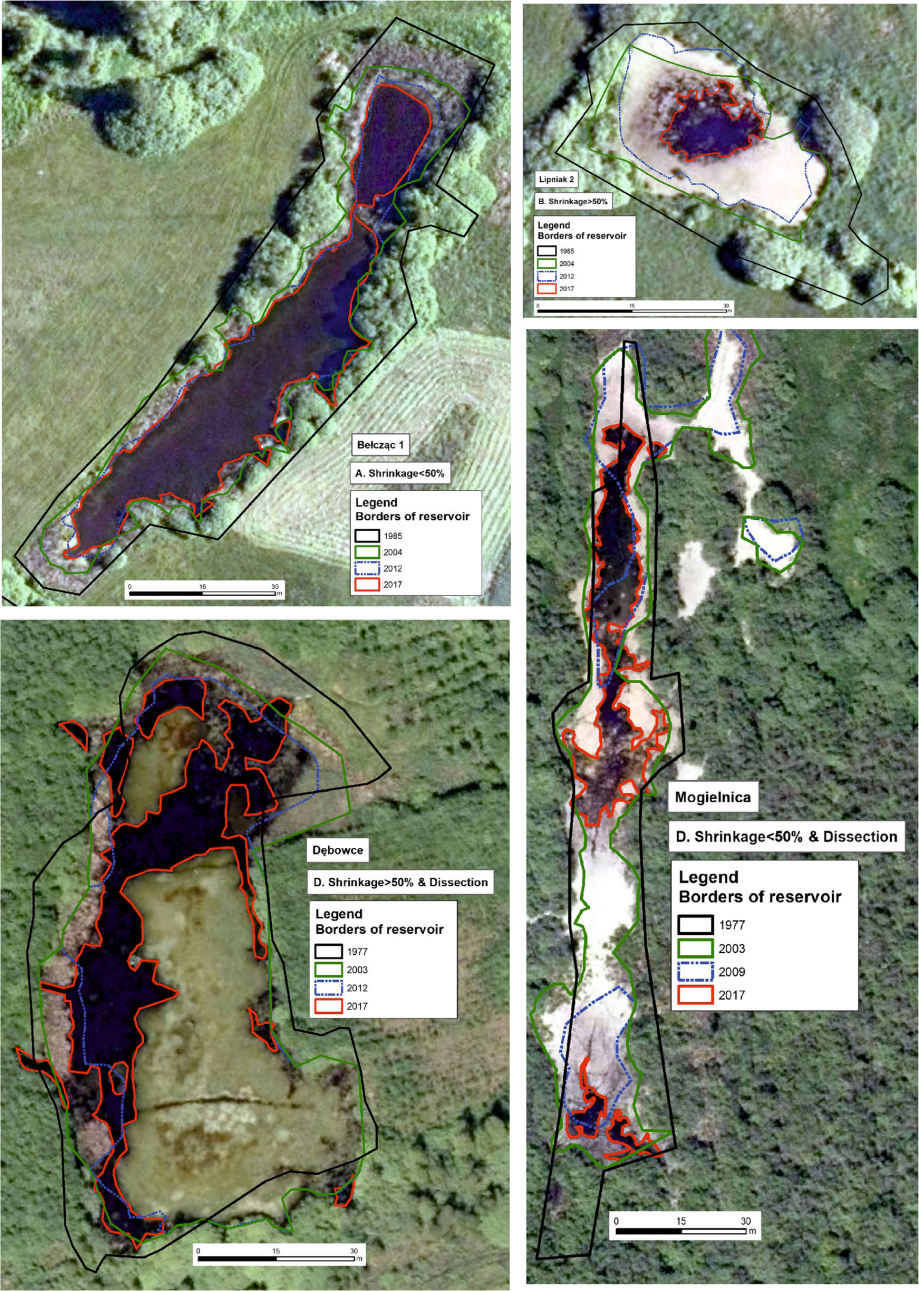
Examples of four models of habitat vanishing based on the selected reservoirs

Generally, four models of reservoir vanishing can be observed. The first one, known as Shrinkage < 50%, is characteristic for relatively steady reservoirs, where the area has been shrunk slightly between P1 and P4 and the water area decreased by less than 50% between P3 and P4 (Fig. 3A). Such a model is typical for 47% of reservoirs. The second model (shrinkage > 50%), which is characteristic for 16% of reservoirs, differs from the previous one as the water area decreased by more than 50% between two last analyzed periods (Fig. 3B). Two other models, apart from the decrease in water area, feature the dissection process—the compact water reservoir was replaced by a few small water bodies, with the total area decreased by less than 50% (Fig. 3C) (25% of reservoirs) or more than 50% between P3 and P4 (Fig. 3D) (12% of reservoirs).

### The vanishing rate

In the case of the 13 analyzed lake minnow sites (50%), the *R*_mean_ oscillates between 1 and 2% of the area per year (Table 1 of the ESM). For 3 sites (11%), this value does not exceed 1% of each area per year and is higher than 2% for the rest of the sites (39%). The similar values were also obtained for individual reservoirs comprising each site. The mean vanishing rate close to 3% of the area per year can be determined in only 9 reservoirs creating larger complexes (site nos. 14, 15, 17, 23, and 25). Different results were obtained based on the *R*_15-years_. Only for three sites, the rate was between 1 and 2% per year, and for 15 sites, it is greater than 4% per year. The highest *R*_15-years_ is recorded for stand nos. 19 (6.63% per year), 7 (6.57% per year), 21 (6.26%), and 24 (6.21% per year). Individual reservoirs comprising each site show quite similar trends.

The ANOVA test (*n* = 52; *α* = 0.05; *F*_crit_ = 4.034) with post hoc Scheffé test showed that the difference in means between the mean and 15-year rate is statistically significant in the case of the analyzed sites (ANOVA: *F*_1,50=_63.5609; *p* < 0.00001). The analysis (*n* = 222; *α* = 0.05; *F*_crit_ = 3.860) also showed a statistical significance in the case of individual reservoirs composing each site (ANOVA: *F*_1,220=_104.3824; *p* < 0.00001). Generally, in the case of 91% of reservoirs, *R*_15-years_ is significantly higher than *R*_mean_ indicating the intensification of overgrowing processes during the last years. Reservoirs which feature a lower value of *R*_15-years_ than *R*_mean_ (9%) belong to a greater complex and the mean rate calculated for those sites still shows the general trend.

### Prognosis year of reservoir vanishing

**Fig. 4:**
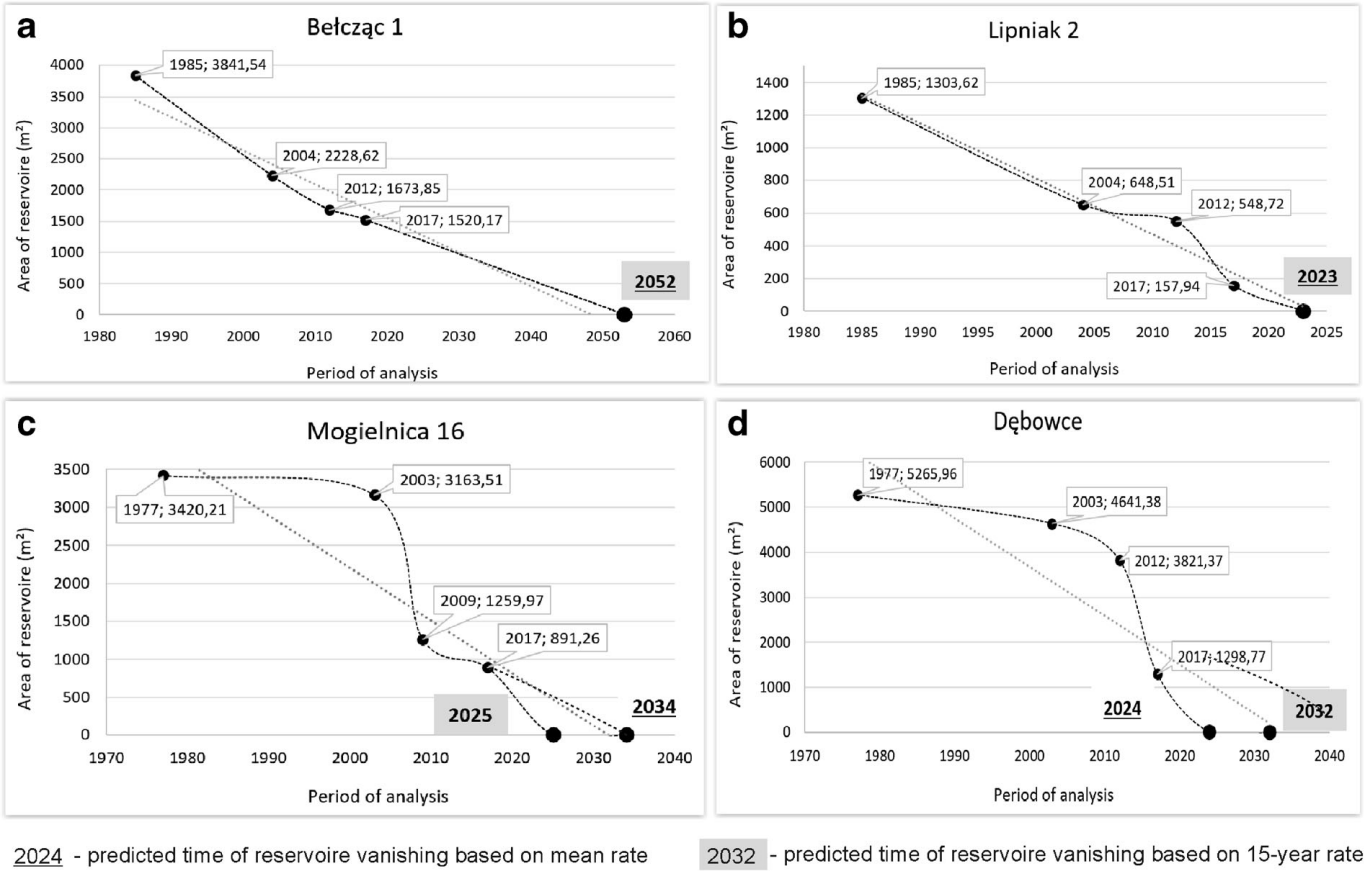
Predicted time of selected reservoirs vanishing based on *R*_mean_ and *R*_15-years_

Prognosis year of reservoir vanishing based on *R*_15-years_ was similar to *R*_mean_ ± 5 years in the case of 47 reservoirs (10 sites) (Fig. 4A, B), and significantly shorter based on *R*_mean_ in the case of 98 reservoirs (13 sites) (Fig. 4C). Only three sites—nos. 3, 14, and 26—would disappear faster based on *R*_mean_ than on *R*_15-years_ according to the prognosis model (Fig. 4D). The ANOVA test showed that the differences in prediction year as to when habitats will vanish based on *R*_15-years_ and *R*_mean_ are statistically insignificant in the case of analyzed sites (ANOVA: *n* = 52; *α* = 0.05; *F*_crit_ = 4.034; *F*_1,50=_0.3328; *p* = 0.5666), as well as in the case of individual reservoirs (ANOVA: *n* = 222; *α* = 0.05; *F*_crit_ = 3.860; *F*_1,220=_0.7087; *p* = 0.4008).

Based on the PYCor_mean_, it was found that if the process of overgrowing and shallowing were not stopped, 15, or 58%, of the analyzed lake minnow sites would disappear in the next 50 years (of which 8 (31%) in the next 20 years). According to PYCor_15-years_, the prognosis is even worse, as 17 peat holes (65%) are due to disappears in the next 50 years (of which 11 (42%) in the next 20 years). Sites Nos. 7, 13, 19, and 21 are particularly vulnerable to extinction. They are predicted to vanish in 2024–2025, 2022–2025, 2020–2021, and 2019–2021, respectively, based on the observed trend. If the current rate of processes continues, site nos. 14 and 18 and 25 and 26 will survive the longest.

### Relationships between the time in which a site is predicted to vanish and its location

**Fig. 5:**
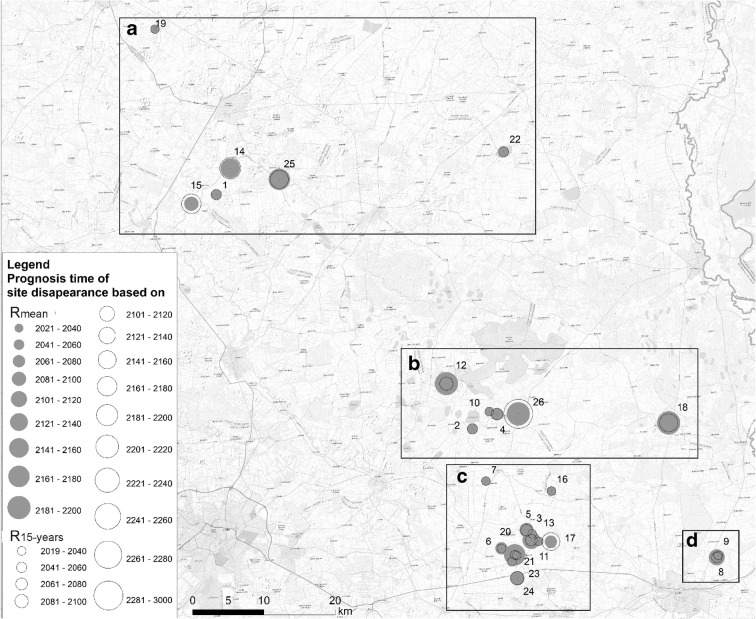
The projected year of analyzed site disappearance based on the *R*_mean_ and *R*_15-years_ in relation with four distinct groups due to their geographical situation: (A) the Tyśmienica river valley; (B) the Polesie region; (C) the Mogielnica and Świnka river valleys; (D) the surrounding of the Gotówka village

The lake minnow sites under analysis are divided into four distinct groups depending on their geographical situation. They include (A) the Tyśmienica river valley (nos. 1, 14, 15, 19, 22, 25); (B) the Polesie region (nos. 2, 4, 10, 12, 18, 26); (C) the Mogielnica and Świnka river valleys (nos. 3, 5, 6, 7, 11, 13, 16, 17, 20, 21, 23, 24); and (D) the surrounding of the Gotówka village (nos. 8, 9) (Fig. 5).

The ANOVA test showed that differences in means between *R*_mean_ and *R*_15-years_ in relation to each site belonging to groups A–D are insignificant (ANOVA: *n* = 52; *α* = 0.05; *F*_crit_ = 2.790; *F*_3,48_ = 0.4829; *p* = 0.6954), but the differences in means between PYCor_15-years_ and PYCor_15-years_ are statistically significant (ANOVA: *n* = 52; *α* = 0.05; *F*_crit_ = 2.790; *F*_3,48_ = 3.4802; *p* = 0.0229). The differences in means in relation to each reservoir are significant both in relation to each site (ANOVA: *n* = 221; *α* = 0.05; *F*_crit_ = 2.623; *F*_3,218_ = 3.5026; *p* = 0.016) and reservoir (ANOVA: *n* = 221; *α* = 0.05; *F*_crit_ = 2.623; *F*_3,218=_ 6.3795; *p* = 0.0004). Tukey’s post hoc tests showed that significant differences are between groups B and C as well as A and C (Table 2). Generally, apart from the quite similar vanishing rate for all groups, which oscillates around 3%, sites from groups A and D are more vulnerable to extinction and reservoirs representing group B will survive the longest according to the prediction model.

**Table 2:** Tukey’s post hoc tests

Treatment pair	Sites		Reservoirs	
	R	PYCor	*R*	PYCor
A vs B	i.s.*	i.s.	i.s.	i.s.
A vs C	i.s.	i.s.	*p* < 0.05	*p* < 0.01
A vs D	i.s.	i.s.	i.s.	i.s.
B vs C	i.s.	*p* < 0.05	i.s.	*p* < 0.01
B vs D	i.s.	i.s.	i.s.	i.s.
C vs D	i.s.	i.s.	i.s.	i.s.

*i.s. means insignificant

### The correctness of the model

**Fig. 6:**
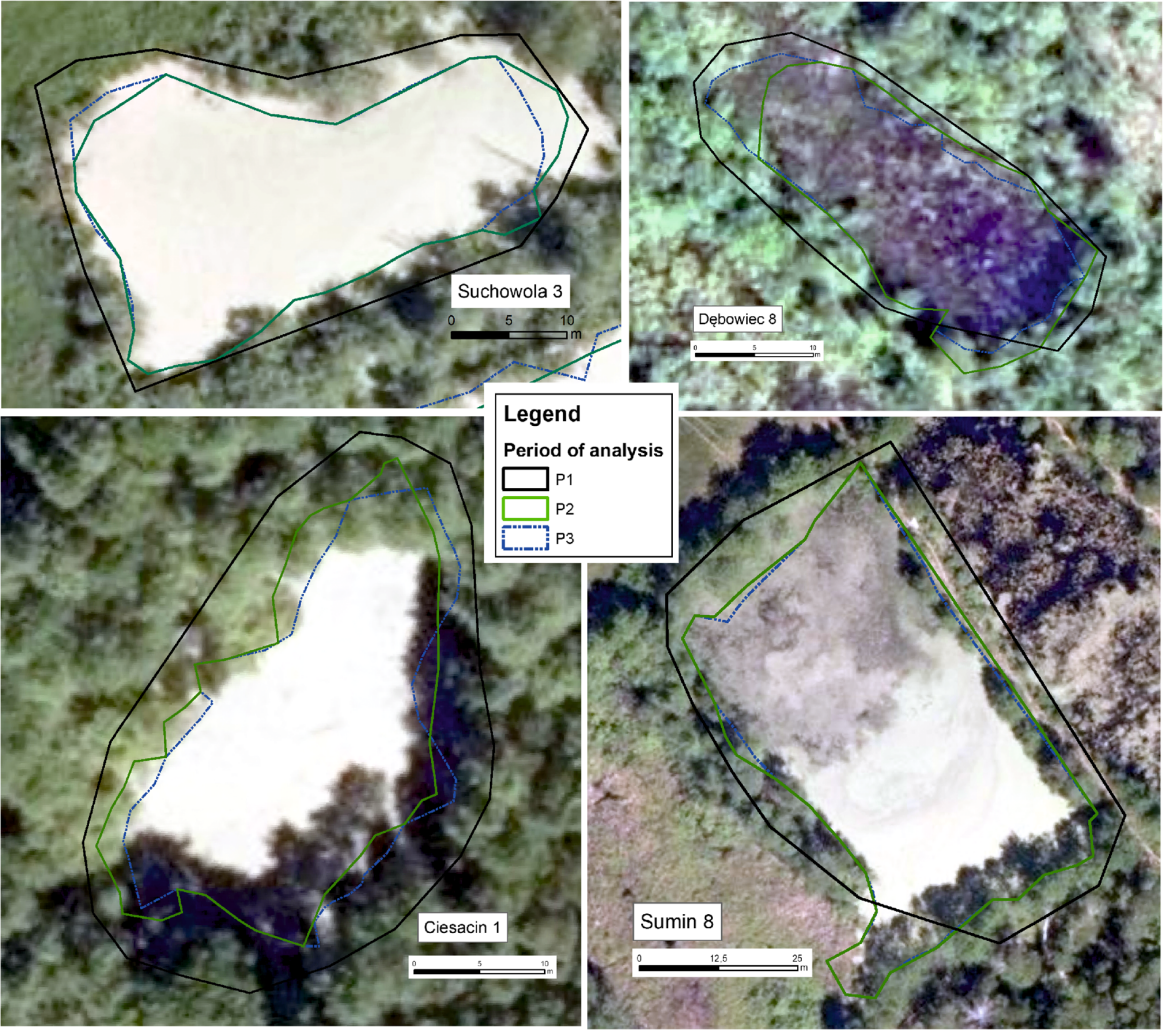
Examples of peat holes that have already disappeared contrary to the prediction model

As mentioned in the “Materials and methods” section, 35 of the analyzed reservoirs were not visible on the latest analyzed period (2017/2018)—they are marked by * in the Supplementary Materials. The prognosis model predicted this situation in reference to 25 reservoirs (71%). In relation to the remaining ten peat holes, the prognosis occurred to be uncorrected—the model showed the longest existence of reservoir with comparison to the state in the year 2018. Surprisingly, apart from one reservoir (no. 2.2.), the prognosis time of vanishing of those remaining nine peat holes was significantly longer (between 2042 and 2262) than the actual one (between 2009/2012 and 2017/2018). The model was unable to prognose this situation as the water area of those nine reservoirs was relatively stable between P1 and P3 (Fig. 6). In the case of 14 peat holes (56%) that already had vanished, the better predictor occurred to be *R*_15-years_. *R*_mean_ predicted the disappearance of seven peat holes (28%) and both rates (*R*_mean_ and *R*_15-years_) of four peat holes (16%). The model, however, 100% correctly predicted the existence of the remaining 76 reservoirs after the P4. Generally, taking into account the state in 2019, the model was corrected in relation to 90% of reservoirs.

## Discussion

### Extinction threats to the Eastern Poland lake minnow population in comparison to other regions

The results clearly indicate that the analyzed water reservoirs inhabited by lake minnow are gradually disappearing. If these processes do not stop, 58% of them will disappear in the next 50 years (of which 8 (31%) in the next 20 years). These findings are generally consistent with the estimations found for other Polish regions. Radke et al. (2011) stated that about 50% of the 103 documented sites in the Pomorskie voivodship are classified as threatened, including 10% endangered at a low level, and 43% at a high level. In the Mazowieckie voivodship, six sites (40%) are presently considered as critically threatened, whereas seven appear to be the least vulnerable (Wolnicki et al. 2011b). Other regions, however, seem to have less endangered sites in comparison to Eastern Poland. Sikorska and Wolnicki (2011) indicated only one site in the Wielkopolskie voivodeship, and Wolnicki et al. (2011c) found two sites in the Kujawsko-Pomorskie voivodeship classified as critically threatened. Generally, at least 75% of habitats of lake minnow are currently in danger of being destroyed (Kaczmarczyk and Wolnicki 2016). In the light of the results, it should be emphasized that the loss of lake minnow habitats is part of a more complex process, which is the loss of the wetland habitats and the resulting decrease in population of most aquatic species. The first symptoms of this process in Eastern Poland were observed in the second half of the twentieth century. The main reason for this regression was that a large part of the wetlands, especially small and shallow reservoirs, has degraded virtually irreversibly. They have disappeared or degraded as a result of them being deliberately dried and changed in cultivation. Lowering of groundwater levels and regulation of rivers have also been contributing factors. These transformations have affected not only the functional area of the Polish Eastern Europe (Kolejko et al. 2007, Kolejko and Wolnicki 2006) but also other regions of the country (Kosturkiewicz et al. 1983, Pieńkowski 1996, Nowicki 1997) and all parts of Europe (Feld et al. 2011). The freshwater ecosystems have degraded severely affecting entire rivers, their catchments, floodplains, and estuaries. It is estimated that in recent decades, in Europe, about one-third of such habitats have disappeared (Hillbricht-Ilkowska and Pieczyńska 1993). As a consequence, global evidence suggests that at least 4% of all known fish species will become extinct in the near future (Bruton 1995, Groombridge and Baillie 1997). Other researchers (Leidy and Moyle 1998) speculate that as many as 20% of fish species will become extinct. Even freshwater megafauna species are threatened globally, with intense and increasing human pressures occurring in many of their biodiversity hotspots. According to the research conducted by He et al. (2018), 71% of megafauna species, including 130 fishes in the total set, are in danger. Ripple et al. (2019) assessed this percentage as relatively lower—between 17.6 and 31.2% depending on the fish group. Nevertheless, all studies suggested that the rate of extinction increases every decade and all representatives of all fish groups are in constant danger.

### Factors affecting the rate at which sites disappear

The dependence—between the rate of disappearance and the site location—clearly showed that two local populations inhabiting the Tyśmienica valley and area of the Gotówka village are more in danger of extinction due to habitat loss than other sites. Analyzing this relation, the authors examined different hypotheses. The first one considers the physical and chemical factors of water. It was assumed that the vanishing rate is related to the amount of electrolytic conductivity and concentration of nutrients. However, analysis of these factors showed no clear relationship. Electrolytic conductivity values for sites located in the Tyśmienica valley and the area of the Gotówka village, with the exception of peripherally located site no. 19, vary between 320 and 471 μS cm^−1^, with the mean value of 421 μS cm^−1^ (Wolnicki and Kolejko 2008). However, the values for sites less threatened with extinction located in the Mogielnica-Świnka river valleys are quite similar as they vary between 339 μS cm^−1^ and 484 μS cm^−1^, with the mean value of 397 μS cm^−1^. Sites located in the Polesie region, however, showed clear differences. They have significantly lower electrolytic conductivity values which vary between 32 and 230 μS cm^−1^, with the mean value amounting to 176 μS cm^−1^. Notwithstanding, the above relationships do not explain clearly the relation between the mean vanishing rate and localization. Moreover, no clear relationship was found in relation to the depth of the reservoirs, which determines the amount of underground water supplied. The depth of peat holes varies between 0.5 and 1.5 m depending on weather conditions independently from their location. Another factor that can affect the rate of lake minnow habitat loss is the degree to which peat bogs are preserved. Drainage causes dehydrated layers of peat to desiccate and rot. The process can lead to nutrients being transported and accumulated, which may consequently accelerate the shallowing. However, even this hypothesis concerning the analyzed sites was not fully confirmed. The results show quite the opposite. The meadows being the analyzed lake minnow habitats in the Tyśmienica valley have not been drained, as opposed to those located in sites in the valley of the Mogielnica and Świnka rivers. Therefore, the authors are not able to provide a clear explanation of the relationship identified between the habitat vanishing rate and their geographical location. However, it should be emphasized that the disappearance of lake minnow habitats in the Tyśmienica valley has been documented since the 1960s. Based on the 1960–1963 data, Danilkiewicz (1965) estimated that there were hundreds of potential lake minnow habitats (peat holes) in the area. The inventory carried out between 2005 and 2008 by Wolnicki and Kolejko (2008) showed, however, that this region has many fewer reservoirs that could become a habitat and only six of them are inhabited by this species.

### Benefits and limitations of the adopted approach

The benefit of the adopted approach lies in the fact that together with the present state of the habitats, its past changes have been analyzed. This knowledge is crucial to observe the species decline and thus to assess the extinction risk (Schrott et al. 2005). Analyzing the data from several decades earlier makes it possible to follow the trajectory of changes and thus predict more precisely whether and when the habitat will vanish. The disturbance history may show that some habitats which currently looks stable in fact are under threat or vice versa: habitats that seem to be vanishing have been in this state for decades, and if any strong disturbance occurred, they would survive (Schrott et al. 2005). Therefore, extinction risk assessment based on a current landscape state only is incomplete, and potentially misleading. Conservation goals should not be planned on this basis only, because this might lead to inadequate or too slow reactions, and thus, the population may vanish. The major limitation of the presented method is the fact that the prediction as to extinction risk inter alia depends on at which point of time this is being assessed, and this depends on the availability of data (Coulson et al. 2001, O’Grady et al. 2004, Klippel et al. 2016). Satellite-based remote sensing is helpful in examining landscape changes over the past three decades. The previous state of the landscape illustrating topographic maps which are not so precise when it comes to the small reservoirs. However, since the majority of the analyzed lake mirror habitats are “young” (about 60 years old), the time between their creation and the earliest available cartographic image is relatively short. Nevertheless, extinction risk prediction may be misleading to some extent or for some sites. In addition, other researchers underline the fact that prediction accuracy should be improved to obtain a more realistic view of fish habitat extinction risk (McCusker et al. 2017). In reference to our study, the model occurred to be incorrect in relation to ten reservoirs, which already vanished that were not determined based on the prediction model. Moreover, apart from one reservoir (no. 2.2.) the PYCor of those peat holes was significantly longer (between 2042 and 2262) than the actual one (between 2009/2012 and 2017/2018). It resulted from the fact that the surface of the water of those peat holes was relatively stable between P1 and P3. The decrease of the water area was ~ 10% between P1 and P2 and 5% between P2 and P3. Site no. 4 (Dębowiec) has already disappeared totally and the model predicts this situation in reference to 82% of peat holes constituting this site. In other cases, however, the source of disturbances is difficult to determine. The authors are aware that to provide a full picture of the disappearance of analyzed species habitats, it would be necessary to delineate its area every year or two. It would eliminate errors resulting from the temporary, local fluctuations, contrary to the general trend, such as flood or drought. However, such an approach is not possible because there are no appropriate cartographic materials.

Finally, any prediction has its probability as only simple parameters are used as extinction risk indicators. Therefore, data are often incomplete even for threatened species (Mace and Lande 1991, Harding et al. 2001). Moreover, the process of extinction is difficult to study mathematically, and consequently, most results in this area are approximations. Besides, apart from the land-use changes, there are lots of predictable and unpredictable factors that determine whether the habitat/population will survive or not (O’Grady et al. 2004). In the case of lake minnow, they include water level fluctuations, restocking reservoirs with species economically attractive to anglers (carp, pike), and presence of alien species (Chinese sleeper dry, brown bullhead) (Wolnicki and Radke 2009, Wolnicki and Kolejko 2008). As a result of competition for nutrients and the predator–prey relation, they can contribute significantly to the decline of individual metapopulations and, in extreme cases, to their total extinction. Given the small depth of the reservoirs, drainage and prolonged droughts can, too, contribute to the total loss of habitat. On the other hand, reverse processes may also occur. Natural water damming brought about by beavers, whose number on the analyzed area is significant, may improve habitat conditions. However, in the case of all aquatic species, if the water reservoir vanishes, their local population will become extinct. Besides, lake minnow habitats are isolated and rarely connected with flowing waters which hinder the movement of individuals.

### Management perspective

The results of the analysis clearly showed that the population of lake minnow in eastern Poland is threatened with extinction. Despite the fact that various forms of nature conservation areas have been created, the number of their habitats has been falling steadily since the 1950s. During this period, about 60% of the sites disappeared only in the West Polesie, a fact documented in the 1960s and 1970s (Wolnicki and Kolejko 2008). To counter these negative processes it is crucial to take immediate action in two directions: activities aimed at maintaining existing sites and restoring the species. The latter actions have been carried out since the autumn of 2013 in the Poleski National Park. Three reservoirs were restocked with 150 individuals from a population of donor site Sumin (Wolnicki 2013). This type of actions are recommended for at least a dozen other reservoirs, where previously there were lake minnow but their habitat conditions are within the tolerance range of this species. Unfortunately, almost all the peat bog holes where the species were reported in the 1950s and 1960s have been significantly transformed and the expected effect of restoration would be difficult to predict.

Establishing protected areas is mostly recommended to maintain habitats of rare fauna species. However, despite the fact that among the analyzed sites 15 (about 60%) of them are located in the area of Natura 2000 Sites (PLB060004, PLB060015, PLB060019, PLH060009, PLH060033, PLH060065, PLB060002, PLH060095), the results of the analysis indicated that they are subject to atrophy to the same degree as other sites that are not protected. Therefore, from the management point of view, the main conclusion resulting from the study is that passive protection is not sufficient to maintain the existing population of lake minnow. It is necessary to take active action, such as the restoration and maintenance of a steady level of groundwater appropriate for the species, maintenance of the water level through damming (wherever it is possible), deepening of a reservoir, and removal of shrubs. Others sites require that garbage and waste deposited in them should be removed. Above all, it is necessary to discontinue drainage works that affect the habitat conditions of some reservoirs. In Natura 2000 sites, such works have not been conducted so far, mainly because the areas have only been protected for a short period of time and because it is necessary to prepare the plans for implementing protective action for selected habitats and species. It should be noted, however, that not all currently existing habitats are classified for passive and active protection. In some cases, it is not possible because of the legal status of private properties (land ownership), in others—because the state in which the habitat is preserved indicates that protective measures will not be effective.

## Conclusions

1. The analysis revealed that in the case of 50% of the analyzed sites, the mean vanishing rate oscillates between 1 and 2% of the area per year and is higher than 2% for 39% of the sites.
2. The prediction model showed that if the trends continue, 58% of analyzed lake minnow habitats, or 37% of all the documented sites in Eastern Poland, will disappear in the next 50 years (of which 8 (31%) in the next 20 years).
3. The model also indicates that the lake minnow population in the Mogielnicy and Świnka valleys and in the Polesie region will survive the longest, whereas the population inhabiting the Tyśmienica valley and the surrounding of the Gotówka village is the most threatened with extinction.
4. Taking into account the state in the year 2019, the model was corrected in relation to 90% of analyzed reservoirs. The prediction was more accurate based on the 15-year rate of vanishing (*R*_15-years_).
5. From the management perspective, the passive protection occurred to be insufficient to preserve the lake minnow habitats, in the case of which active protective action should be immediately undertaken.

## Electronic supplementary material

Table 1 (DOCX 21 kb)
