# Supplementary material for: Extinction risk to lake minnow (Eupallasella percnurus) due to habitat loss: Eastern Poland case study
Source: Environ Monit Assess. 2019 Aug 16;191(9):571. doi: 10.1007/s10661-019-7731-6 (PMC6697757; doi:10.1007/s10661-019-7731-6)
Supplement: Supplementary file 1 — (DOCX 21 kb) [file 10661_2019_7731_MOESM1_ESM.docx]

**Table 1.** Results of risk analysis of the lake minnow habitat loss

|  | Site | Reservoir | R_mean_ | R _15-years_ | PYCor_mean_ | PYCor_15-years_ | PYCor  versus  state at 2017/2018 |
| --- | --- | --- | --- | --- | --- | --- | --- |
| 1 | Bełcząc | 1 | 1,89 | 2,45 | 2052 | 2052 | + |
|  |  | 2 | 2,74 | 6,64 | 2021 | 2019 | + |
|  |  | sum | **2,31** | **4,54** | **2052** | **2052** | **+** |
| 2 | Ciesacin* | 1* | 2,87 | 2,85 | 2024 | 2022 | - |
|  |  | 2* | 2,55 | 3,89 | 2059 | 2056 | - |
|  |  | sum | **2,68** | **3,37** | **2059** | **2056** | - |
| 3 | Dębowce |  | **1,88** | **5,14** | **2024** | **2032** | + |
| 4 | Dębowiec* | 1* | 3,67 | 7,57 | 2024 | 2015 | +  PYCor_15-years_ |
|  |  | 2* | 2,51 | 10,60 | 2015 | 2011 | +  PYCor_mean_  PYCor_15-years_ |
|  |  | 3* | 1,09 | 0,66 | 2032 | 2017 | +  PYCor_15-years_ |
|  |  | 4* | 1,64 | 3,26 | 2022 | 2017 | +  PYCor_15-years_ |
|  |  | 5* | 1,03 | 4,09 | 2065 | 2018 | +  PYCor_15-years_ |
|  |  | 6* | 1,36 | 5,68 | 2048 | 2018 | +  PYCor_15-years_ |
|  |  | 7* | 1,67 | 3,09 | 2042 | 2063 | - |
|  |  | 8* | 1,54 | 1,62 | 2059 | 2062 | - |
|  |  | 9* | 1,62 | 9,00 | 2039 | 2015 | +  PYCor_15-years_ |
|  |  | 10* | 0,42 | 1,45 | 2042 | 2017 | +  PYCor_15-years_ |
|  |  | 11* | 2,40 | 2,89 | 2017 | 2036 | +  PYCor_mean_ |
|  |  | sum | **1,72** | **4,54** | **2065** | **2063** | + |
| 5 | Dobromysl II |  | **0,86** | **1,33** | **2095** | **2080** | + |
| 6 | Gliny north | 1* | 1,78 | 6,41 | 2033 | 2018 | +  PYCor_15-years_ |
|  |  | 2 | 1,15 | 3,62 | 2070 | 2037 | + |
|  |  | 3 | 2,36 | 6,71 | 2025 | 2024 | + |
|  |  | 4 | 1,51 | 4,50 | 2048 | 2030 | + |
|  |  | 5 | 2,78 | 3,29 | 2060 | 2039 | + |
|  |  | 6* | 1,19 | 6,21 | 2057 | 2015 | +  PYCor_15-years_ |
|  |  | sum | **1.80** | **4,58** | **2070** | **2039** | + |
| 7 | Głębokie | 1 | 2,22 | 6,25 | 2025 | 2022 | + |
|  |  | 2 | 2,47 | 6,83 | 2019 | 2020 | + |
|  |  | 3 | 2,42 | 6,63 | 2024 | 2024 | + |
|  |  | sum | **2,37** | **6,57** | **2025** | **2024** | + |
| 8 | Gotówka I | 1 | 0,73 | 2,51 | 2104 | 2049 | + |
|  |  | 2 | 1,27 | 4,82 | 2041 | 2025 | + |
|  |  | sum | **1,00** | **3,66** | **2104** | **2049** | + |
| 9 | Gotówka II |  | **1,13** | **3,90** | **2049** | **2027** | + |
| 10 | Grabniak | 1 | 2,40 | 7,37 | 2021 | 2019 | + |
|  |  | 2 | 1,76 | 5,07 | 2035 | 2025 | + |
|  |  | 3 | 2,39 | 6,78 | 2021 | 2021 | + |
|  |  | sum | **1,92** | **5,78** | **2035** | **2025** | + |
| 11 | Janowica |  | **0,69** | **1,95** | **2130** | **2062** | + |
| 12 | Jelino | 1 | 1,69 | 3,01 | 2041 | 2042 | + |
|  |  | 2 | 1,32 | 1,37 | 2190 | 2083 | + |
|  |  | 3 | 2,37 | 6,81 | 2021 | 2021 | + |
|  |  | 4 | 1,32 | 3,62 | 2059 | 2038 | + |
|  |  | 5 | 1,33 | 2,18 | 2058 | 2056 | + |
|  |  | 6 | 1,75 | 5,30 | 2040 | 2029 | + |
|  |  | sum | **1,63** | **3,46** | **2190** | **2083** | + |
| 13 | Kolonia Zabitek |  | **2,09** | **5,23** | **2025** | **2022** | + |
| 14 | Lichty | 1 | 2,59 | 4,56 | 2028 | 2030 | + |
|  |  | 2 | 0,96 | 1,88 | 2095 | 2063 | + |
|  |  | 3 | 2,30 | 2,40 | 2029 | 2046 | + |
|  |  | 4 | 0,65 | 0,96 | 2145 | 2114 | + |
|  |  | 5 | 1,28 | 0,53 | 2069 | 2197 | + |
|  |  | sum | **1,65** | **2,60** | **2145** | **2197** | + |
| 15 | Lipniak | 1 | 1,93 | 4,67 | 2040 | 2028 | + |
|  |  | 2 | 2,75 | 5,82 | 2023 | 2023 | + |
|  |  | 3 | 1,09 | 0,18 | 2085 | 2177 | + |
|  |  | 4* | 2,57 | 5,04 | 2019 | 2017 | +  PYCor_15-years_ |
|  |  | sum | **2,08** | **3,93** | **2085** | **2177** | + |
| 16 | Ludwinów |  | **2,08** | **4,96** | **2031** | **2029** | + |
| 17 | Mogielnica | 1 | 2,49 | 7,03 | 2021 | 2021 | + |
|  |  | 2 | 2,33 | 6,61 | 2020 | 2018 | + |
|  |  | 3 | 2,04 | 0,15 | 2027 | 2156 | + |
|  |  | 4* | 2,89 | 1,36 | 2016 | 2080 | +  PYCor_mean_ |
|  |  | 5 | 2,20 | 5,36 | 2024 | 2024 | + |
|  |  | 6 | 2,27 | 6,23 | 2021 | 2019 | + |
|  |  | 7* | 1,33 | 4,20 | 2043 | 2018 | +  PYCor_mean_ |
|  |  | 8* | 2,10 | 3,07 | 2017 | 2028 | +  PYCor_mean_ |
|  |  | 9 | 2,16 | 5,42 | 2024 | 2022 | + |
|  |  | 10* | 1,04 | 4,74 | 2066 | 2017 | +  PYCor_15-years_ |
|  |  | 11 | 2,19 | 5,70 | 2022 | 2020 | + |
|  |  | 12 | 2,27 | 5,54 | 2023 | 2023 | + |
|  |  | 13 | 1,56 | 3,71 | 2049 | 2038 | + |
|  |  | 14 | 2,19 | 5,55 | 2023 | 2022 | + |
|  |  | 15* | 1,89 | 4,16 | 2021 | 2018 | +  PYCor_15-years_ |
|  |  | 16 | 1,85 | 5,13 | 2034 | 2025 | + |
|  |  | 17 | 2,38 | 6,21 | 2021 | 2021 | + |
|  |  | 18 | 1,56 | 2,40 | 2049 | 2053 | + |
|  |  | 19 | 2,41 | 5,70 | 2020 | 2022 | + |
|  |  | 20 | 2,47 | 6,77 | 2021 | 2022 | + |
|  |  | sum | **2,08** | **4,76** | **2066** | **2156** | + |
| 18 | Podpakule | 1 | 0,14 | 0,70 | 2192 | 2157 | + |
|  |  | 2 | 0,99 | 1,59 | 2084 | 2078 | + |
|  |  | 3 | 0,16 | 0,71 | 2292 | 2155 | + |
|  |  | 4 | 0,42 | 1,89 | 2222 | 2068 | + |
|  |  | 5 | 1,46 | 4,78 | 2051 | 2036 | + |
|  |  | 6 | 0,22 | 1,05 | 2241 | 2112 | + |
|  |  | sum | **0,57** | **1,79** | **2192** | **2157** | + |
| 19 | Rzymki |  | **2,74** | **6,63** | **2021** | **2020** | + |
| 20 | Siedliszcze I | 1 | 1,11 | 3,04 | 2172 | 2039 | + |
|  |  | 2 | 1,14 | 4,74 | 2065 | 2025 | + |
|  |  | 3 | 1,86 | 5,79 | 2029 | 2018 | + |
|  |  | sum | **1,71** | **5,05** | **2172** | **2039** | + |
| 21 | Siedliszcze II |  | **2,26** | **6,26** | **2021** | **2019** | + |
| 22 | Skoki |  | **1,70** | **2,76** | **2054** | **2050** | + |
| 23 | Stasin north | 1 | 2,10 | 5,70 | 2025 | 2020 | + |
|  |  | 2 | 2,01 | 6,40 | 2028 | 2020 | + |
|  |  | 3 | 2,30 | 6,52 | 2023 | 2021 | + |
|  |  | sum | **2,13** | **6,21** | **2028** | **2021** | + |
| 24 | Stasin south | 1 | 2,02 | 5,24 | 2026 | 2022 | + |
|  |  | 2 | 1,62 | 3,77 | 2045 | 2035 | + |
|  |  | 3* | 2,59 | 9,40 | 2017 | 2015 | +  PYCor_mean_  PYCor_15-years_ |
|  |  | 4* | 2,71 | 10,37 | 2017 | 2016 | +  PYCor_mean_  PYCor_15-years_ |
|  |  | 5* | 2,25 | 8,21 | 2020 | 2014 | +  PYCor_15-years_ |
|  |  | 6* | 1,61 | 3,27 | 2025 | 2016 | +  PYCor_15-years_ |
|  |  | 7 | 1,00 | 1,30 | 2082 | 2085 | + |
|  |  | sum | **1,97** | **5,62** | **2082** | **2085** | + |
| 25 | Suchowola | 1 | 1,14 | 2,07 | 2078 | 2058 | + |
|  |  | 2* | 2,22 | 1,03 | 2018 | 2088 | +  PYCor_mean_ |
|  |  | 3* | 0,56 | 0,30 | 2168 | 2143 | - |
|  |  | 4* | 0,21 | 0,25 | 2107 | 2210 | - |
|  |  | 5* | 0,71 | 0,09 | 2131 | 2126 | - |
|  |  | 6* | 0,21 | 0,24 | 2171 | 2119 | - |
|  |  | 7* | 2,31 | 3,47 | 2017 | 2023 | +  PYCor_mean_ |
|  |  | 8* | 3,15 | 7,75 | 2015 | 2015 | +  PYCor_mean_  PYCor_15-years_ |
|  |  | 9* | 1,35 | 1,33 | 2060 | 2080 | - |
|  |  | 10* | 3,35 | 6,54 | 2017 | 2021 | +  PYCor_mean_ |
|  |  | 11 | 2,25 | 4,47 | 2031 | 2028 | + |
|  |  | sum | **1,59** | **2,50** | **2168** | **2143** | -/_+_ |
| 26 | Sumin | 1 | 1,78 | 4,54 | 2039 | 2032 | + |
|  |  | 2 | 2,48 | 7,52 | 2019 | 2020 | + |
|  |  | 3 | 2,05 | 4,82 | 2027 | 2026 | + |
|  |  | 4 | 1,20 | 0,08 | 2066 | 2299 | + |
|  |  | 5 | 1,32 | 3,64 | 2055 | 2034 | + |
|  |  | 6 | 2,44 | 7,42 | 2020 | 2020 | + |
|  |  | 7 | 2,11 | 6,28 | 2024 | 2020 | + |
|  |  | 8* | 0,11 | 0,40 | 2190 | 2262 | - |
|  |  |  | **1,69** | **4,34** | **2190** | **2299** | -/+ |

*Reservoirs and sites not visible on ortophotomap 2017/2018

+ Means the correctness of prognosis model: model predicts reservoir disappearance or it existence after 2017/2018

- Means the incorrectness of prognosis model: model does not predict reservoir disappearance between 2009/2012 and 2017/018
